# Polyphenols and IUGR Pregnancies: Effects of Maternal Hydroxytyrosol Supplementation on Hepatic Fat Accretion and Energy and Fatty Acids Profile of Fetal Tissues

**DOI:** 10.3390/nu11071534

**Published:** 2019-07-05

**Authors:** Consolación Garcia-Contreras, Marta Vazquez-Gomez, Zaira Pardo, Ana Heras-Molina, José Luis Pesantez, Teresa Encinas, Laura Torres-Rovira, Susana Astiz, Rosa Nieto, Cristina Ovilo, Antonio Gonzalez-Bulnes, Beatriz Isabel

**Affiliations:** 1 SGIT-INIA, Ctra. De La Coruña Km. 7,5, 29040-Madrid, Spain; 2 Faculty of Veterinary Medicine, UCM, Ciudad Universitaria s/n, 28040-Madrid, Spain; 3 Department of Physiology and Biochemistry of Animal Nutrition, Estacion Experimental del Zaidin, Spanish National Research Council (CSIC), Cno.del Jueves s/n, Armilla, 18100 Granada, Spain; 4 School of Veterinary Medicine and Zootechnics, Faculty of Agricultural Sciences, University of Cuenca, Avda. Doce de Octubre, 010220 Cuenca, Ecuador

**Keywords:** antioxidants, intrauterine-growth-restriction, pregnancy, swine-model

## Abstract

Maternal supplementation with hydroxytyrosol, a polyphenol present in olive leaves and fruits, is a highly promising strategy to improve the oxidative and metabolic status of fetuses at risk of intrauterine growth restriction, which may diminish the appearance of low-birth-weight neonates. The present study aimed to determine whether hydroxytyrosol, by preventing lipid peroxidation, may influence the fat accretion and energy homeostasis in the liver, as well as the fatty acid composition in the liver and muscle. The results indicate that hydroxytyrosol treatment significantly decreased the energy content of the fetal liver, without affecting fat accretion, and caused significant changes in the availability of fatty acids. There were significant increases in the amount of total polyunsaturated fatty acids, omega-3 and omega-6, which are highly important for adequate fetal tissue development. However, there were increases in the omega-6/omega-3 ratio and the desaturation index, which make further studies necessary to determine possible effects on the pro/anti-inflammatory status of the fetuses.

## 1. Introduction

Offspring affected by a low-birth-weight (LBW) after suffering processes of intrauterine growth restriction (IUGR) are a concerning issue for both human and veterinary medicine, due to the short-term (increased mortality and morbidity of neonates) and long-term consequences of LBW (decreased growth patterns, health status and performance of individuals). Thus, there is a strong necessity for strategies to alleviate the occurrence and consequences of IUGR.

A possible approach is the use of antioxidant agents. The main cause for IUGR is the inadequate supply of nutrients and oxygen to the fetus, by either placental insufficiency or maternal malnutrition (which also causes placental insufficiency [1,2]). Both placental insufficiency and maternal malnutrition cause hypoxia, and hypoxia increases oxidative stress and aggravates IUGR [3,4]. In turn, IUGR fetuses have a weakened antioxidant defense system [5,6] which exacerbates the condition. Hence, antioxidant agents may be useful to improve the antioxidant/oxidative status during pregnancy.

Polyphenols are potent antioxidants with a double action, scavenging reactive oxygen species and inhibiting their formation, and hence may be useful in compromised pregnancies since they increase the plasma antioxidant capacity [7,8] and therefore diminish placental oxidative stress [9]. However, the study of the possible usefulness and/or risks of polyphenols for reproductive health and pregnancy is only just starting [7]. Our group has recently shown, in a swine model of IUGR [10], the usefulness of a maternal supplementation with hydroxytyrosol, a polyphenol present in olive leaves and fruits [11,12], to improve oxidative and metabolic status of IUGR fetuses [13] and to counteract the appearance of LBW neonates [14].

Previous studies using our swine IUGR model [15] evidenced that maternal undernutrition diminishes antioxidant capacity and increases lipid peroxidation of fetuses from early stages of pregnancy, prejudicing lipids and fatty acid availability at later stages of gestation. Lipids, mainly essential fatty acids, are crucial for adequate fetal development and an excess of lipid peroxidation may compromise their availability to the fetus [6,16], worsening IUGR [17,18], because excessive oxidative stress causes the degradation of fatty acids and mainly of polyunsaturated fatty acids (PUFAs), which produce a variety of aldehydes, alkanals, alkenes, and alkanes of pathological significance [19]. Moreover, fatty acids and specifically PUFAs are involved in the regulation of different enzymes and cytokines and therefore metabolic processes [20]. Moreover, in the case of oxidative stress, metabolic and inflammatory status may be even worsened by a higher oxidation of amino acids, specifically L-arginine, which increases production of nitric oxide (NO). NO is a free radical which main physiological functions but causing pathological (pro-inflammatory) condition in case of excess [21].

Phenolic antioxidants avoid formation of free oxygen radicals by donating hydrogen [19]. In agreement with this premise, fetuses from pregnancies supplemented with hydroxytyrosol have a higher antioxidant capacity. Such higher antioxidant capacity is related to enhancement of the glutathione route. Glutathione is a main antioxidant agent which prevents lipid peroxidation [22] and counteract the DNA hypomethylation induced by oxidative stress [13]. Hypomethylation produces fetal liver steatosis and impaired fatty acids profile by impaired β-oxidation in both liver and muscle, which in turns causes long-term epigenetic metabolic dysregulations [23]. The liver is the largest organ that modulates systemic energy homeostasis [24] and, concomitantly, fat is its major energy reserve [25]. Alterations of hepatic energy homeostasis are also related to long-term epigenetic metabolic dysregulations [26]. Hence, we aimed to determine whether maternal hydroxytyrosol supplementation may influence fat accretion and energy homeostasis in the liver and fatty acid availability in both the muscle and the liver of the offspring.

## 2. Materials and Methods 

### 2.1. Animals and Experimental Procedure

The study was performed according to the Spanish Policy for Animal Protection RD53/2013, which complies with the European Union Directive 2010/63/UE on the protection of animals used for research. The experimental procedures were specifically assessed and approved by the INIA Committee of Ethics in Animal Research (report CEEA 2013/036). Sows were housed at the INIA facilities, which meet local, national and European policy for Scientific Procedure Establishments. 

The study involved 13 pregnant Iberian sows. From gestation Day 1 to 35 their individual daily maintenance requirements were fulfilled with a standard grain-based feed with the following mean nutrient composition: dry matter, 89.8%; crude protein, 15.1%; fat, 2.8%; and metabolizable energy, 3.0 Mcal/kg. Diet analysis showed that the most abundant fatty acids (FA) were palmitic (18.7%), oleic (23.2%) and linoleic acids (46.5%), as detailed in Table 1.

Pregnancy diagnosis was performed at Day 35 after artificial insemination, following estrous synchronization for assuring the same gestational age of all fetuses. Afterwards, the sows were weighed and pair-matched according to body weight. The amount of food offered to each sow was adjusted to 50% of its daily maintenance requirements (1 kg of food per animal and day), which has been previously found to impose a nutritional challenge inducing IUGR in the offspring [10,27]. Seven females remained as untreated control group (group C) whilst six females (group HTX) acted as the treated group by receiving 1.5 mg of hydroxytyrosol per kg of feed per day, so the daily dose of hydroxytyrosol was 1.5 mg. The compound was mixed with the food and food was offered, in both control and treated groups, in individual feeders in order to ensure that each animal ate all its available diet; there were no refusals.

At Day 100 of pregnancy, corresponding to approximately 90% of a 112-day gestation length typical for this breed, the fetuses were obtained in compliance with RD53/2013 and position and sex were assessed. Day 100 of pregnancy was chosen because, from Day 90 onwards, fetal metabolism becomes independent from maternal signals and is highly affected by nutrient availability [28]. A total of 55 and 45 fetuses were obtained, respectively, in the C group (31 female, 56.4%, and 24 male fetuses, 43.6%) and HTX group (26 female, 57.8%, and 19 male fetuses, 42.2%).

### 2.2. Evaluation of Liver Energy and Adiposity and Fat Composition in Liver and Muscle of Fetuses

Gross energy content of the fetal liver was determined in duplicate with an isoperibolic bomb calorimeter (Parr Instrument Co., Moline, IL USA) in freeze-dried homogenized samples from the right lateral lobe. Fat accretion of the liver (in terms of percentage) and fatty acid composition of liver and muscle were determined as previously described [29], in samples from the *longissimus dorsi* muscle and from the right lateral lobe of the liver. Intramuscular and liver fat were extracted from 300 mg of lyophilized and homogenized samples using the Ball-mill procedure [30]. Fatty acids in the total lipid extracts were identified and quantified by gas chromatography (HP6890, Hewlett Packard, Avondale, PA, USA) after methylation as previously described [30,31]. Neutral lipid fractions (triglycerides) and polar lipid fractions (phospholipids) were analyzed using gas chromatography after passing them through aminopropyl minicolumns previously activated with 7.5 mL of hexane [30]. Fatty acid methyl esters were fractionated on a cross-linked polyethylene glycol capillary column (30 cm × 0.32 mm × 0.25 μm, Hewlett Packard Innowax) and a temperature gradient from 170 °C to 245 °C. The injector and detector were maintained at 250 °C. The percentages of individual fatty acids were used to calculate proportions of SFA, MUFA and PUFA, as well as total n-3 and n-6 and their ratio (∑n-6/∑n-3). The unsaturation index (UI) was obtained from the ratio of MUFA to SFA, and the activity of the stearoyl-CoA desaturase enzyme 1 (SCD1) was inferred from the ratio of the enzyme product, oleic acid (C18:1n-9), to the enzyme substrate, stearic acid (C18:0).

### 2.3. Statistical Analysis

Data were analyzed using SPSS^®^ 22.0 (IBM, Armonk, NY, USA). Effects of maternal diet (control vs. treated) and fetal sex (female vs. male) and the interaction sex* treatment on adiposity and fatty-acid composition were assessed by two-way ANOVA. Duncan’s post-hoc test was performed to check differences among groups in multiple comparisons. The sow was considered the experimental unit for all variables in order to avoid biasing the results according to litter size: Fetuses with the same sex from the same sow were averaged together, giving one data point per sow. All results were expressed as mean ± SEM and statistical significance was accepted from *P* < 0.05, while *P*-values between 0.05 and 0.09 were considered to indicate a tendency.

## 3. Results

The assessment of fatty acid composition showed significant and highly consistent effects of the hydroxytyrosol supplementation on gross energy content of the liver and on both the neutral and polar fatty acid fractions of both the liver and the longissimus dorsi muscle.

### 3.1. Effects on Energy and Fat Content of the Liver

Maternal hydroxytyrosol supplementation diminished the gross energy content of the fetal liver when compared to the group C (5162.2 ± 148.3 vs. 5249.5 ± 184.3 cal/g dry matter, respectively; *P* < 0.001). Such difference was driven by fetal sex, with males of group HTX showing lower energy levels than males of group C (5146.2 ± 227.7 vs. 5257.9 ± 303.7 cal/g dry matter, respectively; *P* < 0.05). Conversely, there were no differences in fat content between C and HTX fetuses (13.6 vs. 13.7% of dry matter, respectively).

### 3.2. Effects on Fatty Acid Composition of Liver

Maternal hydroxytyrosol supplementation was related to very few common changes in the neutral and polar fractions of the fetal liver (Table 2 and Table 3); there was only a lower proportion of SFA and similar increases in eicosenoic (C20:1n-9) and adrenic (C22:4n-6) acids. Main changes were found only at the polar fraction where HTX supplementation increased PUFA (with a decrease also in MUFA), Σn-3, Σn-6 and UI, and the relative contents of gamma-linolenic (C18:3n-6), arachidonic (C20:4n-6), adrenic and docosapentaenoic (C22:5n-3) and docohexaenoic (C22:6n-3) acids. On the other hand, the content of palmitic (C16:0), cis-vaccenic (C18:1n-7), palmitoleic (C16:1n-7), heptadecanoic (C17:1) and oleic (C18:1n-9) acids was lower in treated fetuses. The neutral fraction showed higher activity of stearoyl-CoA desaturase enzyme 1 (SCD1), lower content of stearic acid (C18:0) and higher content of cis-7 hexadecenoic (C16:1n-9), oleic, linoleic, eicosenoic and adrenic acids. Other fatty acids which content was increased in treated fetuses were myristic (C14:0), palmitic and palmitoleic acids. Finally, these fetuses showed a higher MUFA content and UI than controls. 

These changes were similar in both sexes, with an interaction sex-treatment for the polar fraction, but with no effects on the neutral fraction. At the polar fraction, hydroxytyrosol decreased the amount of SFA in males, whilst females showed a lowered UI and SCD1 activity.

### 3.3. Effects on Fatty Acid Composition of Longissimus Dorsi Muscle

Maternal hydroxytyrosol supplementation was related, at both neutral and polar fractions of the fetal *longissimus dorsi* muscle (Table 4 and Table 5), with a significant decrease in SFA and significant increases in PUFA, Σn-3, Σn-6, Σn-6/Σn-3 ratio and UI. Specifically, fetuses from sows supplemented with hydroxytyrosol showed, at both neutral and polar fractions, a lower content of stearic and oleic acids and higher content of gamma-linolenic, adrenic and docosapentaenoic) acids than control fetuses. The neutral fraction also showed a significantly higher activity of SCD1 and higher contents of cis-7 hexadecenoic, linoleic (C18:2n-6) and eicosenoic acids in fetuses from supplemented sows. The polar fraction of these fetuses had higher contents of myristic, arachidonic and eicosapentaenoic acids (C20:5n-3) and lower palmitic, heptadecanoic, cis-vaccenic and linolenic acids.

These changes in both neutral and polar fractions were similar in both sexes and there were no major significant differences between males and females within the same treatment group. Interactions between sex and treatment were more evident in treated males, which showed significant increases in the content of PUFA, Σn-3, Σn-6 and UI at the neutral fraction and Σn-3 at the polar fraction.

## 4. Discussion

The results of the present study indicate that the administration of hydroxytyrosol to pregnant sows induces significant changes on the energy content and the fatty acid composition in fetal tissues. 

The maternal hydroxytyrosol supplementation decreases, in a sex-dependent way, the energy content of the liver, with treated male fetuses showing a lower energy content than their control male counterparts. Energy reserves are mostly constituted by fat [25], but there were no differences in the total hepatic fat content between groups. Hence, the difference in energy content may be more related to differences in protein and/or carbohydrate contents; a hypothesis that we cannot support with the data from the current study, and which makes necessary the development of further research. In any case, we have to highlight that in our previous studies on the effects of hydroxytyrosol supplementation we found that the liver, in both fetuses [13] and neonates [14], were smaller in treated offspring, especially in males, than in controls. Such findings, plus the current results of lower energy content, support the need for further studies on possible deleterious effects of hydroxytyrosol on the male liver development.

The main effects of hydroxytyrosol supplementation on the fatty acid composition were found at both the neutral and polar fractions of the longissimus dorsi muscle, where the amount of SFA was decreased whilst the amounts of PUFA, Σn-3 and Σn-6, the ratio Σn-6/Σn-3 and the unsaturation index (UI) were increased. The liver also showed a decrease of SFA at both the neutral and polar fractions but, conversely to the *longissimus dorsi*, there were few main effects on the neutral fraction. Main changes were found at the polar fraction where, similarly to the muscle, hydroxytyrosol increased PUFA, Σn-3, Σn-6 and UI.

The neutral fraction of fatty acids is mainly composed of triglycerides, which are an essential energy source for the fetus [32], while the polar fraction is composed by phospholipids, which constitute cell membranes and are essential for tissue development [32]. Our results indicate that maternal hydroxytyrosol supplementation modifies the polar fraction of fatty acids protecting PUFA from oxidation (i.e., intervenes in the development of cells and tissues, and therefore the organ) in both muscle and liver. However, its effects on the neutral fraction (i.e., in energy partitioning) are mostly restricted to the muscle. A possible explanation for these stronger effects of maternal hydroxytyrosol supplementation on the muscle than on the liver may be related to the fact that, in case of compromised fetal nutrition, as in the present study, the growth of essential organs (brain, liver) is protected at the expenses of other organs [33,34] and mainly at the expense of muscle development [35,36,37]. Hence, muscle development would be more challenged than liver development and the effects of hydroxytyrosol supplementation would be more evident.

Fetal availability of fatty acids is more dependent on its own synthesis from precursors transferred by the mother than on direct maternal transfer [38,39,40,41,42]. However, essential fatty acids (EFAs), which are required for adequate fetal tissue development and pregnancy success [43], must be obtained from maternal diet, since animals are unable to synthesize them. The most important EFAs are polyunsaturated fatty acids (PUFAs); both short-chain PUFA (linolenic acid, an Σn-3 FA, and linoleic acid, an Σn-6 FA) and long-chain PUFA (eicosapentaenoic and docosahexaenoic acids, Σn-3 FA, and gamma-linolenic and arachidonic acids, Σn-6 FA). 

The results obtained in the current trial show that maternal hydroxytyrosol supplementation increases the amounts of total PUFA and total Σn-3 and Σn-6 in both lipid fractions of the muscle and the polar fraction of the liver. Moreover, in our study, EFAs were also specifically increased by the maternal hydroxytyrosol treatment; the amounts of linolenic, gamma-linolenic and arachidonic acids were increased in the polar fractions of both muscle and liver, while linoleic, eicosapentaenoic and also gamma-linolenic acids were increased in the neutral fraction of muscle and docosahexaenoic acid was increased in the polar fraction of the liver. There were no major effects of fetal sex on these changes, which were modulated by the fetal sex only in the case of the muscle neutral fraction.

The higher availability of EFAs in fetuses from pregnancies with maternal hydroxytyrosol supplementation may be related to the antioxidant effects triggered by the treatment, since our previous data indicated that offspring exposed to hydroxytyrosol have increased antioxidant activity [14], which decreases lipid peroxidation and increases its availability. 

A higher availability of omega-6 fatty acids in developing fetuses was is related to critical changes during vascular development [44], while a higher availability of Σn-3 or omega-3 FA may improve insulin function, pro-/anti-inflammatory status and physical and mental development during the first years of life [45,46,47,48]. In fact, as previously described, it is currently recognized that fatty acids (specifically PUFAs) are relevant for the early immune development and maturation by regulating the gene expression of different enzymes and cytokines and numerous metabolic processes [20]. Specifically, a higher availability of omega-3 PUFAs (docosahexaenoic and eicosapentaenoic acids) is related to a higher availability of anti-inflammatory lipids mediators which reduce pathological risks [49,50]. Conversely, a higher availability of Σn-6 or Σn-6 PUFAs (gamma-linolenic and arachidonic acids) is related to a higher availability of pro-inflammatory lipids mediators [51]; which it is not necessarily negative for pregnancy since mid-stage requires anti-inflammatory cytokines for uterine quiescence and optimum fetal growth, but earlier and later stages of pregnancy requires an increased production of pro-inflammatory cytokines for pregnancy establishment and labor stimulation, respectively [52]. However, these data, in addition to other emerging evidence of prolonged gestations and increased birth weights, also support the necessity of establishing appropriate doses of omega-3 and omega-6 PUFAs in different stages of pregnancy [52].

In this sense, our study evidences the positive effects of hydroxytyrosol supplementation on the availability of omega-3 and omega-6 PUFAs but also gives a warning on their adequate balance, addressing the necessity of further studies to evaluate the safety of the treatment proposed. Hydroxytyrosol supplementation increases the ratio Σn-6/Σn-3 and the unsaturation index (UI) at both neutral and polar fractions of the fetal longissimus dorsi muscle and the UI at the neutral fraction of the liver. An excessively increased omega-6 to omega-3 ratio may induce a pro-inflammatory stage, as described in the previous paragraph, while an excessively high UI is currently considered as a potential biomarker of metabolic risk [53,54]. Moreover, UI correlates with the activity of fatty acids desaturases and mainly with SCD1, the enzyme that catalyses the conversion of saturated to monounsaturated fatty acids. Increased SCD1 activity has been related to metabolic disorders; mainly alterations in lipogenesis and insulin regulation in both adult humans [53,54,55,56] and pigs [57,58]. Fetal sex modulated the UI and the activity of SCD1 at the polar fraction of the liver which, suggesting a more protective effect, were decreased in treated females when compared to controls.

In conclusion, the data from the present study evidence that maternal hydroxytyrosol supplementation changes significantly the energy content of the liver and the fatty acid profile of the fetal tissues. Changes in fatty acids profile were mainly driven by increases in the amount of total PUFAs, omega-3 and omega-6, with increases also in the ratio omega-6/omega-3 and the unsaturation index (UI) at both the neutral and polar fractions of the longissimus dorsi muscle and the polar fraction of the liver. These results give preliminary preclinical evidence that maternal hydroxytyrosol supplementation, or consumption of hydroxytyrosol-rich food like olive oil, may be beneficial, on the whole, for improving the metabolic status of fetuses and, specifically, for IUGR-compromised pregnancies. These results may be of clinical relevance but there is not yet enough evidence for counseling its clinical use. In fact, there is a scarcity of experimental data on both the realistic benefits and potential hazards of hydroxytyrosol supplementation during gestation, like with other polyphenols. Hence, additional studies on the safety of polyphenol consumption during pregnancy should be therefore performed prior to its application. Specifically, for hydroxytyrosol, the possible consequences of high omega-6/omega-3 ratio and UI make further studies necessary for determining possible deleterious effects on the pro/anti-inflammatory status of fetuses.

## Figures and Tables

**Table 1:** Fatty acid composition (g/100 g total fatty acids) of the experimental diet.

**Fatty Acid**	**C14:0**	**C16:0**	**C16:1n-9**	**C16:1n7**	**C17:0**	**C17:1**	**C18:0**	**C18:1n-9**	**C18:1n-7**
**Percentage**	0.488	18.722	0.173	0.578	0.540	0.126	3.706	23.201	0.906
**Fatty Acid**	**C18:2n-6**	**C18:3n-3**	**C20:1n-9**	**C20:4n-6**	**C20:5n-3**	**C22:1n-9**	**C22:5n-3**	**C22:6n-3**	
**Percentage**	46.492	3.355	0.648	0.000	0.123	0.268	0.297	0.377	

**Table 2:** Effects and differences between sexes, of maternal hydroxytyrosol supplementation on the fatty acid profile of the fetal liver. Mean values (±SEM) of neutral lipids in female (F) and male (M) fetuses of the treated (HTX) and the control group (C).

	HTX			C			*p*-Value				
Fatty Acids (g/100 g Total Fatty Acids)	Mean	Female	Male	Mean	Female	Male	HTX vs. C Global	Same Sex (HTX vs. C)		Same Experimental Group (F vs. M)	
								HTX vs. C Females	HTX vs. C Males	F vs. M within HTX Group	F vs. M within C Group
**C14:0**	2.98 ± 0.09	2.88 ± 0.09	3.09 ± 0.18	2.50 ± 0.07	2.55 ± 0.10	2.43 ± 0.10	0.000	0.020	0.001	0.192	0.384
**C16:0**	24.98 ± 0.27	24.78 ± 0.31	25.12 ± 0.51	24.13 ± 0.39	24.24 ± 0.48	24.1 ± 0.65	0.086	0.307	0.170	0.339	0.861
**C16:1n-9**	1.88 ± 0.06	1.89 ± 0.08	1.83 ± 0.12	1.33 ± 0.05	1.40 ± 0.07	1.26 ± 0.06	0.000	0.000	0.000	0.815	0.135
**C16:1n-7**	8.20 ± 0.17	7.87 ± 0.19	8.59 ± 0.30	7.23 ± 0.11	7.39 ± 0.17	7.04 ± 0.14	0.000	0.060	0.000	0.025	0.126
**C17:0**	1.23 ± 0.04	1.26 ± 0.06	1.22 ± 0.07	1.31 ± 0.04	1.29 ± 0.05	1.32 ± 0.06	0.172	0.537	0.170	0.519	0.742
**C17:1**	1.09 ± 0.02	1.09 ± 0.03	1.10 ± 0.04	1.06 ± 0.02	1.06 ± 0.02	1.05 ± 0.02	0.210	0.380	0.380	0.753	0.601
**C18:0**	14.87 ± 0.37	15.32 ± 0.41	14.39 ± 0.67	19.41 ± 0.48	18.77 ± 0.69	20.28 ± 0.63	0.000	0.000	0.000	0.190	0.121
**C18:1n-9**	17.79 ± 0.35	17.61 ± 0.47	17.83 ± 0.58	15.61 ± 0.40	15.91 ± 0.55	15.37 ± 0.59	0.000	0.011	0.004	0.767	0.508
**C18:1n-7**	7.52 ± 0.14	7.36 ± 0.14	7.79 ± 0.25	7.26 ± 0.07	7.18 ± 0.10	7.36 ± 0.11	0.077	0.307	0.134	0.155	0.235
**C18:2n-6**	2.89 ± 0.07	2.92 ± 0.08	2.84 ± 0.13	2.65 ± 0.08	2.67 ± 0.10	2.59 ± 0.12	0.022	0.057	0.199	0.363	0.601
**C18:3n-3**	0.24 ± 0.01	0.23 ± 0.01	0.25 ± 0.01	0.23 ± 0.01	0.22 ± 0.01	0.23 ± 0.01	0.173	0.372	0.290	0.367	0.499
**C20:1n-9**	0.34 ± 0.01	0.33 ± 0.01	0.35 ± 0.02	0.27 ± 0.01	0.28 ± 0.02	0.27 ± 0.02	0.000	0.007	0.004	0.500	0.813
**C20:3n-6**	0.38 ± 0.01	0.38 ± 0.01	0.37 ± 0.02	0.37 ± 0.01	0.37 ± 0.02	0.37 ± 0.02	0.954	0.900	0.960	0.613	0.892
**C20:4n-6**	10.83 ± 0.35	11.17 ± 0.41	10.54 ± 0.64	11.68 ± 0.57	11.66 ± 0.72	11.55 ± 0.93	0.221	0.447	0.337	0.311	0.924
**C20:5n-3**	0.21 ± 0.03	0.24 ± 0.05	0.17 ± 0.01	0.20 ± 0.01	0.20 ± 0.02	0.20 ± 0.02	0.869	0.704	0.539	0.569	0.897
**C22:4n-6**	0.61 ± 0.03	0.59 ± 0.04	0.62 ± 0.04	0.50 ± 0.02	0.50 ± 0.03	0.49 ± 0.04	0.004	0.066	0.024	0.55	0.873
**C22:5n-3**	0.39 ± 0.02	0.39 ± 0.02	0.39 ± 0.03	0.36 ± 0.03	0.37 ± 0.04	0.35 ± 0.04	0.516	0.995	0.347	0.606	0.654
**C22:6n-3**	3.59 ± 0.13	3.72 ± 0.15	3.52 ± 0.26	3.89 ± 0.23	3.92 ± 0.31	3.75 ± 0.34	0.287	0.415	0.492	0.364	0.710
**SFA**	44.06 ± 0.21	44.23 ± 0.26	43.81 ± 0.35	47.35 ± 0.61	46.86 ± 0.76	48.13 ± 0.98	0.000	0.004	0.001	0.538	0.302
**MUFA**	36.82 ± 0.63	36.14 ± 0.75	37.49 ± 1.14	32.77 ± 0.56	33.22 ± 0.81	32.34 ± 0.76	0.000	0.006	0.000	0.296	0.441
**PUFA**	19.12 ± 0.54	19.63 ± 0.63	18.7 ± 1.03	19.88 ± 0.91	19.92 ± 1.16	19.53 ± 1.46	0.497	0.680	0.581	0.329	0.832
**UI**	1.14 ± 0.02	1.16 ± 0.02	1.13 ± 0.04	1.15 ± 0.04	1.15 ± 0.05	1.13 ± 0.06	0.903	0.890	0.962	0.414	0.727
**PUFAn-3**	4.42 ± 0.15	4.57 ± 0.18	4.33 ± 0.29	4.68 ± 0.25	4.72 ± 0.34	4.53 ± 0.38	0.408	0.513	0.607	0.411	0.712
**PUFAn-6**	14.7 ± 0.40	15.06 ± 0.47	14.37 ± 0.74	15.2 ± 0.66	15.2 ± 0.83	14.99 ± 1.09	0.540	0.758	0.577	0.309	0.881
**Σn-6/Σn-3**	3.37 ± 0.04	3.33 ± 0.05	3.39 ± 0.07	3.46 ± 0.11	3.46 ± 0.17	3.48 ± 0.15	0.483	0.611	0.637	0.626	0.954
**C18:1/C18:0**	1.80 ± 0.09	1.69 ± 0.09	1.93 ± 0.18	1.23 ± 0.05	1.29 ± 0.07	1.15 ± 0.05	0.000	0.001	0.000	0.200	0.119
**MUFA/SFA**	0.84 ± 0.02	0.82 ± 0.02	0.86 ± 0.03	0.7 ± 0.01	0.71 ± 0.02	0.68 ± 0.02	0.000	0.000	0.000	0.298	0.159

FA (g/100 g Total FA) = Fatty Acids (g/100 g Total Fatty Acids); SFA = Saturated fatty acids. Includes: C14:0, C16:0, C17:0 and C18:0; MUFA = Monounsaturated fatty acids. Includes: C16:1n-9, C16:1n-7, C17:1, C18:1n-9, C18:1n-7, C20:1n-9; PUFA = Polyunsaturated fatty acids. Includes: C18:2n-6, C18:3n-3, C20:3n-9, C20:3n-6, C20:4n-6, C20:5n-3, C22:4n-6, C22:5n-6, C22:5n-3 and C22:6n-3; UI = Unsaturation index; Includes: C18:2n-6, C20:4n-6 and C22:4n-6; Includes: C18:3n-3, C20:5n-3 and C22:6n-3.

**Table 3:** Effects and differences between sexes, of maternal hydroxytyrosol supplementation on the fatty acid profile of the fetal liver. Mean values (±SEM) of polar lipids in female (F) and male (M) fetuses of the treated (HTX) and the control group (C).

	HTX			C			*p*-Value				
Fatty Acids (g/100 g Total Fatty Acids)	Mean	Female	Male	Mean	Female	Male	HTX vs. C Global	Same Sex (HTX vs. C)		Same Experimental Group (F vs. M)	
								HTX vs. C Females	HTX vs. C Males	F vs. M within HTX Group	F *vs*. M within C Group
**C14:0**	1.97 ± 0.04	1.93 ± 0.04	2.06 ± 0.10	2.09 ± 0.05	2.08 ± 0.07	2.07 ± 0.08	0.089	0.038	0.806	0.132	0.767
**C16:0**	23.17 ± 0.18	23.02 ± 0.22	23.33 ± 0.30	24.29 ± 0.40	24.00 ± 0.48	24.72 ± 0.69	0.020	0.082	0.127	0.204	0.340
**C16:1n-9**	1.28 ± 0.020	1.30 ± 0.03	1.27 ± 0.05	1.31 ± 0.03	1.34 ± 0.04	1.27 ± 0.04	0.532	0.534	0.796	0.406	0.316
**C16:1n-7**	4.74 ± 0.08	4.64 ± 0.10	4.94 ± 0.18	5.22 ± 0.10	5.26 ± 0.13	5.14 ± 0.11	0.000	0.001	0.172	0.07	0.472
**C17:0**	1.39 ± 0.04	1.41 ± 0.06	1.37 ± 0.06	1.33 ± 0.04	1.33 ± 0.06	1.32 ± 0.06	0.300	0.387	0.590	0.602	0.823
**C17:1**	0.69 ± 0.01	0.69 ± 0.02	0.69 ± 0.03	0.78 ± 0.02	0.79 ± 0.03	0.77 ± 0.02	0,000	0.015	0.006	0.489	0.547
**C18:0**	19.72 ± 0.20	19.92 ± 0.25	19.37 ± 0.37	20.09 ± 0.26	19.8 ± 0.39	20.55 ± 0.25	0.280	0.614	0.006	0.131	0.113
**C18:1n-9**	10.65 ± 0.14	10.69 ± 0.2	10.47 ± 0.26	11.55 ± 0.27	11.61 ± 0.35	11.64 ± 0.40	0.007	0.108	0.027	0.443	0.778
**C18:1n-7**	6.82 ± 0.10	6.75 ± 0.10	7.13 ± 0.40	7.25 ± 0.14	7.23 ± 0.09	7.14 ± 0.14	0.019	0.026	0.424	0.234	0.495
**C18:2n-6**	2.4 ± 0.05	2.43 ± 0.06	2.36 ± 0.10	2.33 ± 0.04	2.35 ± 0.05	2.29 ± 0.05	0.269	0.276	0.697	0.174	0.311
**C18:3n-3**	0.19 ± 0.00	0.19 ± 0.01	0.20 ± 0.01	0.21 ± 0.01	0.21 ± 0.01	0.20 ± 0.01	0.342	0.221	0.989	0.600	0.460
**C20:1n-9**	0.46 ± 0.01	0.46 ± 0.01	0.46 ± 0.02	0.41 ± 0.01	0.42 ± 0.01	0.41 ± 0.02	0.001	0.015	0.041	0.633	0.648
**C20:3n-6**	18.35 ± 0.19	18.48 ± 0.21	18.13 ± 0.38	16.14 ± 0.54	16.36 ± 0.66	15.79 ± 0.93	0.001	0.006	0.032	0.485	0.572
**C20:4n-6**	0.25 ± 0.01	0.24 ± 0.01	0.25 ± 0.01	0.22 ± 0.01	0.22 ± 0.01	0.21 ± 0.01	0.008	0.118	0.029	0.615	0.585
**C20:5n-3**	0.06 ± 0.00	0.06 ± 0.0	0.07 ± 0.00	0.06 ± 0.00	0.06 ± 0.01	0.06 ± 0.00	0.372	0.986	0.045	0.220	0.679
**C22:4n-6**	0.86 ± 0.04	0.82 ± 0.05	0.91 ± 0.07	0.62 ± 0.03	0.65 ± 0.04	0.59 ± 0.04	0.000	0.009	0,000	0.200	0.367
**C22:5n-3**	0.62 ± 0.02	0.61 ± 0.02	0.63 ± 0.03	0.52 ± 0.03	0.53 ± 0.04	0.49 ± 0.04	0.002	0.102	0.008	0.234	0.358
**C22:6n-3**	6.36 ± 0.10	6.35 ± 0.10	6.37 ± 0.19	5.60 ± 0.23	5.76 ± 0.28	5.35 ± 0.39	0.006	0.090	0.032	0.853	0.335
**SFA**	46.25 ± 0.12	46.27 ± 0.15	46.13 ± 0.25	47.79 ± 0.52	47.21 ± 0.64	48.66 ± 0.83	0.010	0.243	0.014	0.847	0.139
**MUFA**	24.64 ± 0.23	24.53 ± 0.24	24.95 ± 0.55	26.52 ± 0.4	26.65 ± 0.55	26.37 ± 0.6	0.000	0.002	0.046	0.597	0.737
**PUFA**	29.1 ± 0.27	29.2 ± 0.27	28.92 ± 0.53	25.69 ± 0.81	26.14 ± 0.99	24.97 ± 1.4	0.000	0.009	0.018	0.713	0.439
**UI**	1.32 ± 0.01	1.32 ± 0.01	1.32 ± 0.02	1.20 ± 0.03	1.22 ± 0.04	1.17 ± 0.05	0.001	0.027	0.015	0.892	0.343
**PUFAn-3**	7.24 ± 0.10	7.22 ± 0.10	7.27 ± 0.19	6.38 ± 0.25	6.56 ± 0.30	6.09 ± 0.43	0.004	0.087	0.022	0.678	0.305
**PUFAn-6**	21.86 ± 0.19	21.98 ± 0.2	21.65 ± 0.38	19.31 ± 0.57	19.58 ± 0.70	18.88 ± 0.99	0.000	0.003	0.019	0.457	0.516
**Σn-6/Σn-3**	3.04 ± 0.03	3.06 ± 0.03	3.00 ± 0.06	3.11 ± 0.05	3.05 ± 0.06	3.21 ± 0.08	0.233	0.679	0.054	0.326	0.078
**C18:1/C18:0**	0.89 ± 0.02	0.88 ± 0.02	0.92 ± 0.04	0.95 ± 0.02	0.97 ± 0.04	0.91 ± 0.02	0.066	0.040	0.894	0.321	0.235
**MUFA/SFA**	0.53 ± 0.01	0.53 ± 0.01	0.54 ± 0.01	0.56 ± 0.01	0.57 ± 0.01	0.54 ± 0.01	0.019	0.016	0.635	0.578	0.092

FA (g/100 g Total FA) = Fatty Acids (g/100 g Total Fatty Acids); SFA = Saturated fatty acids. Includes: C14:0, C16:0, C17:0 and C18:0; MUFA = Monounsaturated fatty acids. Includes: C16:1n-9, C16:1n-7, C17:1, C18:1n-9, C18:1n-7, C20:1n-9; PUFA = Polyunsaturated fatty acids. Includes: C18:2n-6, C18:3n-3, C20:3n-9, C20:3n-6, C20:4n-6, C20:5n-3, C22:4n-6, C22:5n-6, C22:5n-3 and C22:6n-3; UI = Unsaturation index; Includes: C18:2n-6, C20:4n-6 and C22:4n-6; Includes: C18:3n-3, C20:5n-3 and C22:6n-3.

**Table 4:** Effects and differences between sexes, of maternal hydroxytyrosol supplementation on the fatty acid profile of the fetal *longissimus dorsi* muscle. Mean values (±SEM) of neutral lipids in female (F) and male (M) fetuses of the treated (HTX) and the control group (C).

	HTX			C			*p*-Value				
Fatty Acids (g/100 g Total Fatty Acids)	Mean	Female	Male	Mean	Female	Male	HTX vs. C Global	Same Sex (HTX vs. C)		Same Experimental Group (F vs. M)	
								HTX vs. C Females	HTX vs. C Males	F vs. M within HTX Group	F vs. M within C Group
**C14:0**	3.30 ± 0.05	3.25 ± 0.05	3.29 ± 0.05	3.31 ± 0.09	3.25 ± 0.07	3.24 ± 0.06	0.436	0.65	0.521	0.841	0.923
**C16:0**	29.89 ± 0.20	29.85 ± 0.21	29.85 ± 0.25	29.93 ± 0.34	29.79 ± 0.3	29.93 ± 0.31	0.905	0.875	0.99	0.841	0.748
**C16:1n-9**	2.93 ± 0.13	2.60 ± 0.04	2.91 ± 0.16	2.96 ± 0.24	2.66 ± 0.04	2.52 ± 0.06	0.012	0.118	0.056	0.874	0.05
**C16:1n-7**	6.54 ± 0.15	6.84 ± 0.13	6.55 ± 0.18	6.52 ± 0.27	6.85 ± 0.18	6.83 ± 0.18	0.125	0.248	0.352	0.918	0.921
**C17:0**	1.34 ± 0.04	1.37 ± 0.03	1.33 ± 0.05	1.37 ± 0.05	1.38 ± 0.04	1.35 ± 0.04	0.608	0.414	0.795	0.618	0.572
**C17:1**	1.20 ± 0.04	1.15 ± 0.04	1.22 ± 0.06	1.16 ± 0.06	1.12 ± 0.06	1.20 ± 0.05	0.482	0.424	0.658	0.476	0.37
**C18:0**	11.13 ± 0.12	12.38 ± 0.14	11.00 ± 0.13	11.32 ± 0.22	12.21 ± 0.16	12.58 ± 0.25	0,000	0,000	0.001	0.196	0.202
**C18:1n-9**	22.62 ± 0.15	23.06 ± 0.15	22.89 ± 0.20	22.25 ± 0.23	22.98 ± 0.21	23.16 ± 0.23	0.048	0.752	0.009	0.04	0.574
**C18:1n-7**	7.22 ± 0.09	7.43 ± 0.06	7.11 ± 0.11	7.38 ± 0.14	7.35 ± 0.08	7.52 ± 0.09	0.056	0.081	0.38	0.14	0.13
**C18:2n-6**	4.50 ± 0.11	3.78 ± 0.11	4.60 ± 0.15	4.35 ± 0.17	3.75 ± 0.18	3.82 ± 0.12	0,000	0.001	0.012	0.295	0.764
**C18:3n-3**	0.32 ± 0.01	0.31 ± 0.01	0.32 ± 0.01	0.32 ± 0.02	0.30 ± 0.01	0.32 ± 0.01	0.405	0.302	0.984	0.813	0.378
**C20:1n-9**	0.46 ± 0.01	0.43 ± 0.01	0.45 ± 0.01	0.47 ± 0.01	0.43 ± 0.01	0.42 ± 0.02	0.035	0.34	0.05	0.176	0.677
**C20:3n-6**	0.58 ± 0.01	0.49 ± 0.01	0.57 ± 0.02	0.58 ± 0.02	0.51 ± 0.02	0.47 ± 0.02	0,000	0.04	0,000	0.866	0.163
**C20:4n-6**	5.17 ± 0.17	4.64 ± 0.23	5.12 ± 0.21	5.24 ± 0.29	4.85 ± 0.35	4.38 ± 0.29	0.081	0.529	0.046	0.742	0.316
**C20:5n-3**	0.19 ± 0.01	0.19 ± 0.01	0.19 ± 0.01	0.20 ± 0.01	0.20 ± 0.01	0.18 ± 0.01	0.917	0.275	0.172	0.26	0.188
**C22:4n-6**	1.27 ± 0.03	1.03 ± 0.05	1.26 ± 0.03	1.28 ± 0.04	1.07 ± 0.07	0.97 ± 0.05	0,000	0.025	0,000	0.678	0.238
**C22:5n-3**	0.45 ± 0.01	0.36 ± 0.02	0.46 ± 0.01	0.45 ± 0.02	0.40 ± 0.03	0.32 ± 0.03	0.001	0.126	0.001	0.858	0.114
**C22:6n-3**	0.89 ± 0.02	0.83 ± 0.04	0.88 ± 0.03	0.91 ± 0.04	0.87 ± 0.05	0.79 ± 0.04	0.195	0.879	0.059	0.524	0.249
**SFA**	45.67 ± 0.20	46.84 ± 0.27	45.47 ± 0.24	45.93 ± 0.32	46.64 ± 0.38	47.10 ± 0.39	0.001	0.015	0.031	0.252	0.399
**MUFA**	40.97 ± 0.21	41.51 ± 0.24	41.13 ± 0.25	40.74 ± 0.34	41.4 ± 0.36	41.65 ± 0.30	0.093	0.555	0.052	0.35	0.606
**PUFA**	13.37 ± 0.28	11.64 ± 0.42	13.39 ± 0.37	13.33 ± 0.42	11.96 ± 0.64	11.25 ± 0.53	0.002	0.068	0.005	0.911	0.406
**UI**	0.85 ± 0.01	0.80 ± 0.02	0.85 ± 0.01	0.85 ± 0.02	0.82 ± 0.02	0.79 ± 0.02	0.009	0.185	0.014	0.942	0.316
**PUFAn-3**	1.86 ± 0.03	1.70 ± 0.06	1.84 ± 0.04	1.88 ± 0.05	1.77 ± 0.10	1.61 ± 0.08	0.042	0.523	0.011	0.61	0.213
**PUFAn-6**	11.51 ± 0.25	9.95 ± 0.36	11.55 ± 0.33	11.45 ± 0.38	10.19 ± 0.55	9.64 ± 0.45	0.001	0.047	0.005	0.845	0.456
**Σn-6/Σn-3**	6.20 ± 0.08	5.90 ± 0.1	6.27 ± 0.10	6.10 ± 0.12	5.78 ± 0.14	6.05 ± 0.14	0.025	0.009	0.795	0.257	0.201
**C18:1/C18:0**	2.70 ± 0.04	2.48 ± 0.03	2.74 ± 0.04	2.64 ± 0.06	2.49 ± 0.03	2.46 ± 0.05	0,000	0,000	0.017	0.16	0.515
**MUFA/SFA**	0.9 ± 0.01	0.89 ± 0.01	0.91 ± 0.01	0.89 ± 0.01	0.89 ± 0.01	0.89 ± 0.01	0.207	0.114	0.862	0.162	0.797

FA (g/100 g Total FA) = Fatty Acids (g/100 g Total Fatty Acids); SFA = Saturated fatty acids. Includes: C14:0, C16:0, C17:0 and C18:0; MUFA = Monounsaturated fatty acids. Includes: C16:1n-9, C16:1n-7, C17:1, C18:1n-9, C18:1n-7, C20:1n-9; PUFA = Polyunsaturated fatty acids. Includes: C18:2n-6, C18:3n-3, C20:3n-9, C20:3n-6, C20:4n-6, C20:5n-3, C22:4n-6, C22:5n-6, C22:5n-3 and C22:6n-3; UI = Unsaturation index; Includes: C18:2n-6, C20:4n-6 and C22:4n-6; Includes: C18:3n-3, C20:5n-3 and C22:6n-3.

**Table 5:** Effects and differences between sexes, of maternal hydroxytyrosol supplementation on the fatty acid profile of the fetal longissimus dorsi muscle. Mean values (±SEM) of polar lipids in female (F) and male (M) fetuses of the treated (HTX) and the control group (C).

	HTX			C			*p*-Value				
Fatty Acids (g/100 g Total Fatty Acids)	Mean	Female	Male	Mean	Female	Male	HTX *vs*. C Global	Same Sex (HTX *vs*. C)		Same Experimental Group (F vs. M)	
								HTX *vs*. C Females	HTX *vs*. C Males	F *vs*. M within HTX Group	F *vs*. M within C Group
**C14:0**	2.50 ± 0.03	2.30 ± 0.03	2.45 ± 0.03	2.57 ± 0.05	2.31 ± 0.04	2.30 ± 0.04	0.000	0.009	0.000	0.036	0.784
**C16:0**	23.7 ± 0.11	24.50 ± 0.19	23.59 ± 0.16	23.87 ± 0.13	24.26 ± 0.25	24.8 ± 0.28	0.001	0.034	0.008	0.206	0.157
**C16:1n-9**	2.07 ± 0.05	2.04 ± 0.05	2.02 ± 0.05	2.14 ± 0.10	2.09 ± 0.09	1.97 ± 0.05	0.671	0.479	0.116	0.241	0.267
**C16:1n-7**	3.73 ± 0.08	3.87 ± 0.08	3.63 ± 0.09	3.86 ± 0.14	3.86 ± 0.12	3.87 ± 0.08	0.215	0.152	0.923	0.174	0.938
**C17:0**	1.29 ± 0.03	1.30 ± 0.03	1.29 ± 0.04	1.29 ± 0.05	1.31 ± 0.04	1.30 ± 0.03	0.781	0.781	0.930	0.947	0.895
**C17:1**	0.91 ± 0.03	1.05 ± 0.04	0.89 ± 0.03	0.95 ± 0.06	1.00 ± 0.05	1.11 ± 0.06	0.12	0.078	0.080	0.379	0.223
**C18:0**	11.96 ± 0.08	12.66 ± 0.13	12.10 ± 0.11	11.76 ± 0.11	12.57 ± 0.18	12.78 ± 0.18	0.000	0.038	0.000	0.036	0.418
**C18:1n-9**	22.88 ± 0.13	23.82 ± 0.18	22.96 ± 0.19	22.78 ± 0.16	23.75 ± 0.26	23.92 ± 0.25	0.000	0.021	0.001	0.503	0.644
**C18:1n-7**	6.50 ± 0.06	6.92 ± 0.09	6.46 ± 0.08	6.54 ± 0.11	6.83 ± 0.11	7.02 ± 0.14	0.000	0.010	0.012	0.570	0.281
**C18:2n-6**	5.23 ± 0.11	5.00 ± 0.07	5.32 ± 0.12	5.11 ± 0.19	4.99 ± 0.10	5.01 ± 0.09	0.058	0.036	0.612	0.345	0.865
**C18:3n-3**	0.19 ± 0.01	0.22 ± 0.01	0.19 ± 0.01	0.20 ± 0.01	0.23 ± 0.01	0.20 ± 0.01	0.017	0.004	0.899	0.283	0.082
**C20:1n-9**	0.19 ± 0.01	0.19 ± 0.00	0.19 ± 0.01	0.20 ± 0.01	0.19 ± 0.01	0.19 ± 0.01	0.829	0.626	0.381	0.141	0.859
**C20:3n-6**	0.85 ± 0.01	0.78 ± 0.02	0.86 ± 0.01	0.85 ± 0.03	0.79 ± 0.02	0.78 ± 0.02	0.001	0.012	0.055	0.752	0.818
**C20:4n-6**	13.17 ± 0.11	11.14 ± 0.33	13.25 ± 0.10	13.07 ± 0.21	11.5 ± 0.47	10.69 ± 0.46	0.000	0.001	0.000	0.400	0.234
**C20:5n-3**	0.30 ± 0.01	0.27 ± 0.01	0.30 ± 0.01	0.31 ± 0.01	0.28 ± 0.01	0.26 ± 0.01	0.017	0.358	0.009	0.539	0.238
**C22:4n-6**	1.85 ± 0.02	1.54 ± 0.06	1.84 ± 0.02	1.86 ± 0.03	1.59 ± 0.08	1.48 ± 0.08	0.000	0.004	0.000	0.48	0.326
**C22:5n-3**	0.78 ± 0.01	0.63 ± 0.03	0.77 ± 0.01	0.78 ± 0.02	0.66 ± 0.04	0.58 ± 0.04	0.000	0.026	0.000	0.784	0.18
**C22:6n-3**	1.89 ± 0.02	1.77 ± 0.04	1.90 ± 0.03	1.86 ± 0.04	1.80 ± 0.05	1.74 ± 0.06	0.018	0.088	0.116	0.371	0.508
**SFA**	39.46 ± 0.13	40.77 ± 0.27	39.43 ± 0.18	39.49 ± 0.17	40.45 ± 0.37	41.17 ± 0.40	0.000	0.023	0.001	0.802	0.192
**MUFA**	36.28 ± 0.19	37.89 ± 0.26	36.14 ± 0.24	36.47 ± 0.30	37.73 ± 0.38	38.09 ± 0.35	0.000	0.001	0.002	0.396	0.503
**PUFA**	24.26 ± 0.16	21.34 ± 0.48	24.43 ± 0.19	24.03 ± 0.27	21.82 ± 0.69	20.74 ± 0.67	0.000	0.001	0.000	0.229	0.272
**UI**	1.27 ± 0.01	1.17 ± 0.02	1.27 ± 0.01	1.26 ± 0.01	1.18 ± 0.03	1.14 ± 0.03	0.000	0.003	0.000	0.331	0.25
**PUFAn-3**	3.16 ± 0.03	2.89 ± 0.08	3.16 ± 0.04	3.15 ± 0.05	2.97 ± 0.11	2.79 ± 0.11	0.003	0.122	0.007	0.874	0.246
**PUFAn-6**	21.1 ± 0.15	18.46 ± 0.41	21.27 ± 0.17	20.88 ± 0.25	18.86 ± 0.59	17.96 ± 0.57	0.000	0.000	0.000	0.194	0.284
**Σn-6/Σn-3**	6.70 ± 0.06	6.45 ± 0.07	6.74 ± 0.08	6.64 ± 0.10	6.41 ± 0.11	6.50 ± 0.10	0.012	0.019	0.305	0.417	0.56
**C18:1/C18:0**	2.46 ± 0.02	2.43 ± 0.02	2.44 ± 0.03	2.50 ± 0.03	2.44 ± 0.02	2.43 ± 0.03	0.305	0.955	0.096	0.191	0.731
**MUFA/SFA**	0.92 ± 0.01	0.93 ± 0.01	0.92 ± 0.01	0.92 ± 0.01	0.93 ± 0.01	0.93 ± 0.01	0.263	0.194	0.884	0.645	0.504

FA (g/100 g Total FA) = Fatty Acids (g/100 g Total Fatty Acids); SFA = Saturated fatty acids. Includes: C14:0, C16:0, C17:0 and C18:0; MUFA = Monounsaturated fatty acids. Includes: C16:1n-9, C16:1n-7, C17:1, C18:1n-9, C18:1n-7, C20:1n-9; PUFA = Polyunsaturated fatty acids. Includes: C18:2n-6, C18:3n-3, C20:3n-9, C20:3n-6, C20:4n-6, C20:5n-3, C22:4n-6, C22:5n-6, C22:5n-3 and C22:6n-3; UI = Unsaturation index; Includes: C18:2n-6, C20:4n-6 and C22:4n-6; Includes: C18:3n-3, C20:5n-3 and C22:6n-3.
